# The “inverted-U” of cognitive effort across speech rates holds for simple but not complex sentence structures

**DOI:** 10.3389/fpsyg.2025.1685938

**Published:** 2025-10-29

**Authors:** Ryan M. O’Leary, Nataniela Zavlun, Alex Kinney, Arthur Wingfield

**Affiliations:** Department of Psychology, Volen National Center for Complex Systems, Brandeis University, Waltham, MA, United States

**Keywords:** pupillometry, effort, time-compression, inverted-U, syntactic complexity

## Abstract

Pupil dilation has become a well-established physiological index of cognitive effort. Here we examined the often-cited finding of an inverted U-shaped function of pupil size across the continuum from highly degraded to clear speech. Unlike the short sentences with simple syntax as used in the original inverted-U demonstrations, 32 young adults recalled sentences with subject-relative embedded clause structures and more syntactically complex object-relative embedded clause structures. Sentences were degraded using time-compression and presented at 10, 25, 35 and 100% of their normal playing time selected from a preliminary calibration study. Subject-relative sentences showed an approximately symmetrical inverted U-shaped function across speech rates, but object-relative sentences failed to show the drop in pupil size with normal-rate speech. We conclude that the symmetric inverted U-shaped function of pupil size with increasing speech quality is limited to sentences with simple syntax. We thus offer a more complete picture of the inverted-U than currently in the literature.

## Introduction

Although varying in detail from one language to another, all societies on earth have developed a rich speech system to communicate complex thoughts and intentions. In the everyday world, however, it is rare for spoken language to be heard under ideal listening conditions. In addition to the rapidity of natural speech that can challenge limits on processing speed (Chodorow, 1979; Foulke, 1971), speech quality is frequently degraded by extrinsic sources such as the presence of background noise (Peelle and Wingfield, 2022; Zekveld et al., 2011), informational masking from competing speakers (Ohlenforst et al., 2018), or intrinsic sources, such as hearing impairment (Kuchinsky et al., 2014; Zekveld et al., 2011).

In spite of these challenges, comprehension of spoken languages is noted for its resilience. However, even successful comprehension of rapid or degraded speech requires cognitive effort (recruitment of resources) on the part of the listener (Rönnberg et al., 2013; see also Ayasse et al., 2021; Peelle, 2017; Pichora-Fuller et al., 2016). A fundamental question central to speech communication is thus how best to characterize the relationship between speech quality, cognitive effort, and performance.

It should be noted that the term, *effort*, is often modified as listening effort, cognitive effort, or more generally, processing effort, depending on context or author. In the remainder of this article, we adopt a generic definition of effort as offered in the Framework for Understanding Effortful Listening (FUEL; Pichora-Fuller et al., 2016); “The deliberate allocation of mental resources to overcome obstacles in goal pursuit when carrying out a task.” We use the terms, *cognitive effort*, or *processing eff*ort, as inclusive of listening effort attendant to the recruitment of resources required for successful processing of a degraded acoustic signal at the perceptual level (Pichora-Fuller et al., 2016) and processing at the linguistic level when the stimuli consist of meaningful sentences (Carpenter et al., 1994; DeCaro et al., 2016).

Pupillometry (the measurement of task-related dilation of the pupil of the eye) has become a well-established physiological index of cognitive effort (Kuchinsky and Milvae, 2023). For example, one can observe dilation of the pupil while listeners are attempting to understand speech degraded by acoustic masking (Ohlenforst et al., 2018), degraded by a mild-to-moderate hearing loss (Kuchinsky et al., 2014; Zekveld et al., 2011), and processing especially rapid speech (O’Leary et al., 2023, 2025a). An increase in pupil dilation also appears while listeners are processing sentences that express their meaning with complex syntax (Ayasse and Wingfield, 2018; Just and Carpenter, 1993; Piquado et al., 2010).

Although a monotonic relationship between pupil dilation and increasing perceptual or cognitive demands has been reliably demonstrated, it has been shown that this relationship holds only within limits. Norman and Bobrow (1975) (see also Knight et al., 2023) offered two terms that are useful in an attempt to characterize this non-linear relationship between task difficulty and the effort likely to be allocated to a task.

At one extreme are occasions where speech quality is so impoverished by background noise, accelerated speech rates, or other acoustic perturbations, that successful recognition appears nearly impossible (a *data-limited process*). In the framework of behavioral economics, individuals are unlikely to expend effort on a task when there is an unlikely return on investment (Eckert et al., 2016). This would result in, or approach, a floor effect in performance, and a relatively smaller pupillary response as an indicator of this reduced effort.

As the quality of the speech improves, one sees the often-cited monotonic relationship between speech quality, effort, and performance; in Norman and Bobrow’s terms, a *resource-limited process*. As the quality of the speech further improves, the pupillary response becomes smaller due to the task becoming easier, hence requiring less effort.

**Figure 1:**
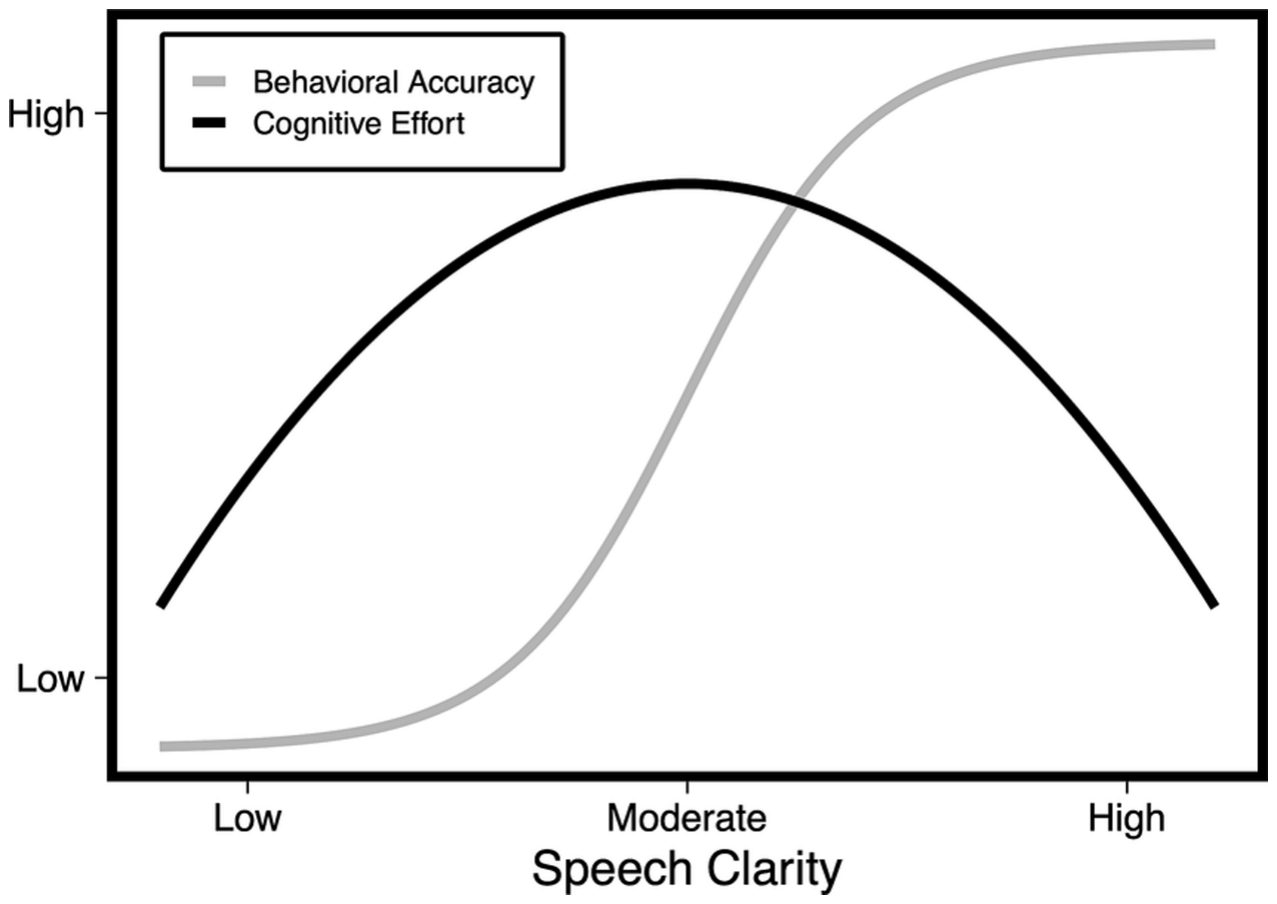
Idealized representation of postulated effort as a function of speech clarity (black curve) superimposed on the proportion of speech materials reported correctly (gray curve).

The above principles are illustrated in an idealized form in Figure 1. One curve shows a typical sinusoidal psychometric function representing performance accuracy as a function of speech clarity. Superimposed on this performance curve is a second curve that represents cognitive effort, showing an inverted U-shaped function as speech quality improves (see Kuchinsky and Milvae, 2023, p. 237 for a similar function).

Figure 1 is an idealized schematic as it excludes effects of task demands that impact cognitive resources such as speed/accuracy tradeoffs (O’Leary et al., 2025b), and pupillary dynamics beyond the acoustic clarity of the stimulus, such as attention to the task and the listener’s motivation to succeed (cf., Pichora-Fuller et al., 2016; Richter, 2016).

The postulated inverted U-shaped function in pupil dilation nevertheless appears as a reliable finding (Ohlenforst et al., 2017, 2018; Wendt et al., 2018) and has been frequently cited in cognitive hearing science (see, for example, Kuchinsky and Milvae, 2023; Keur-Huizinga et al., 2024).

The focus of the present study is on the region of asymptotic performance, where Ohlenforst et al. (2018) and Wendt et al. (2018) have shown a drop in pupillary-indexed processing effort that approximates the level observed for a data-limited process. Because the interest in such studies has been on perceptual effort in the face of acoustic degradation, the stimuli have focused on short, syntactically simple sentences such as the 5-word active-declarative sentences from Nielsen and Dau’s (2009) Danish Hearing in Noise Test (HINT) (Ohlenforst et al., 2017, 2018; Wendt et al., 2018). It is possible, however, that the observed drop in pupillary response in the asymptotic region of task performance is limited to the use of syntactically simple sentences with which the drop has been observed. That is, it may be that additional difficulty introduced at the linguistic level may carry over to influence the shape of the inverted-U function. The following experiment was designed to test this hypothesis, offering a more complete picture of the performance-effort relationship than currently represented in the literature.

Two sentence types were chosen for this test: sentences with a subject-relative center-embedded clause (*SR* sentences) versus syntactically more complex object-relative center-embedded clauses (*OR* sentences).

Sentences with an SR structure (e.g., “Girls with big black backpacks that leave boys are harsh”) take a canonical form in which the first noun in the sentence (*girls*) is the agent that performs an action (*leave boys*), with the action the first verb encountered, and the recipient of the action (boys) is the second noun encountered. Sentences with an SR structure were contrasted with syntactically more complex OR sentences (e.g., “Boys that girls with big black backpacks leave are harsh”). It can be seen that the meaning has not changed (girls still leave boys), but the structure departs from the canonical form of an SR sentence.

A critical feature of the SR versus OR contrast is that they can be constructed to contain exactly the same words, albeit in a different order. In this way, factors such as word frequency and the number of phonological competitors for each word are automatically equated. Importantly, it is already well established in the psycholinguistic literature that OR sentences produce more errors in comprehension and recall than SR sentences (Carpenter et al., 1994; DeCaro et al., 2016; Obler et al., 1991; Stewart and Wingfield, 2009; Wingfield et al., 2006). Pertinent to the present study, it has also been shown that even with clear speech in quiet conditions, processing OR sentences results in relatively larger pupil dilations than SR sentences (Ayasse et al., 2021; Just and Carpenter, 1993; Piquado et al., 2010).

To examine whether the well-documented inverted-U pattern of effort generalizes beyond acoustic masking, we incorporated time-compressed speech to parametrically vary processing time and perceptual demands, allowing us to examine how speech rate and syntactic challenge jointly influence the effort–performance relationship.

## The present experiment

The following experiment was conducted in two stages using time-compression to control speech rate. The first was a calibration study to establish the psychometric function representing a continuum of recall accuracy for SR and OR sentences as a function of decreasing degrees of time-compression. These data would be used to select compression ratios of interest for pupillometric analysis in the main experiment.

Manipulating performance accuracy with time-compression has the interesting feature that at moderate compression ratios, the difficulty is primarily due the loss of ordinarily available processing time, while at very high compression ratios a loss of richness of the speech signal is a limiting factor in phonological and lexical identification (Chodorow, 1979; Heiman et al., 1986; Wingfield et al., 1999).

The main experiment was conducted with a larger group of participants who were tested for recall of SR and OR sentences at four compression ratios selected to represent low, moderate, and high recall performance as determined in the calibration study. In this main experiment pupil dilation was recorded as an index of processing effort. Reducing the number of compression ratios allowed an increase in the number of trials per speech rate to increase power without lengthening the duration of the experiment and risking participant fatigue, a concern when using pupillometry (Winn et al., 2018).

Based on the previously cited studies that have demonstrated an inverted U-shaped function (e.g., Ohlenforst et al., 2017, 2018; Wendt et al., 2018), we expect to see a smaller pupil size in the lower end of the performance curve, larger pupil sizes when performance was at approximately 50%, and, for the simpler SR sentences, a drop in pupil size when accuracy has reached a performance asymptote. In the case of OR sentences, the cognitive effort attendant to processing these more complex sentences may counter the traditional drop in processing effort at the asymptotic performance level.

## Methods

### Calibration study: determining the performance curves for SR and OR sentences

#### Participants

Ten young adults, 4 males and 6 females, aged 18–26 years (*M* = 19.4, SD = 1.58) took part in the calibration study. Participants’ hearing was tested using a Grason-Stadler AudioStar Pro clinical audiometer (Grason-Stadler, Inc., Madison, WI) by way of standard audiometric procedures in a sound-attenuated testing room. All participants had age-normal hearing, with a pure tone average (PTA) across 0.5, 1, 2, and 4 kHz of 5.00 dB HL (hearing level) (SD = 4.04). All participants reported English as their first language. Written informed consent was obtained for a protocol approved by the Brandeis University Institutional Review Board (IRB).

#### Stimuli

Creation of the stimuli began with the construction of 140, 9-word grammatical English sentences with an SR structure (e.g., “Girls with coffee mugs that approach guys are social”). These are referred to as base sentences. For each SR base sentence, an OR sentence was created by changing the word order to yield a sentence with the same meaning, but expressed with an OR construction (e.g., “Girls with coffee mugs that approach guys are social”). This process produced a total of 280 test sentences. In addition to the test sentences, 50 filler sentences without a relative clause were included to discourage participants from potentially recognizing patterns within the stimuli. Performance on the 50 filler sentences was not analyzed.

Both test sentences and fillers were recorded by a male speaker of American English using natural prosody onto computer sound files using Sound Studio v2.2.4 (Felt Tip, Inc., New York, NY) that digitized (16-bit) at a sampling rate of 44.1 kHz. Recordings were equalized across sentences for root-mean-square (RMS) intensity using Praat (Boersma and Weenink, 2022).

Recordings of each of the 280 test sentences (140 SR sentences; 140 OR sentences) and the 50 filler sentences were time-compressed to be reproduced in 10, 15, 20, 25, 30, 35, 40, 60, 80, and 100% (not compressed) of the original playing time, corresponding to 1,486, 977, 743, 586, 495, 419, 371, 244, 186, and 146 words per minute (wpm). We note here that the increments were intentionally uneven, as we wished to sample critical regions along the speech rate continuum. To put these figures in perspective, everyday conversational speech averages between 140 to 180 wpm, while a radio or TV newsreader working from a prepared script may reach 210 wpm.

Time-compression was conducted using Praat (Boersma and Weenink, 2022) employing the pitch-synchronous overlap-and-add (PSOLA) algorithm. This compression algorithm uses uniform linear compression such that both words and silent pauses were reduced equivalently to maintain the relative temporal pattern of the original recordings. The algorithm leaves the original pitch contour intact.

#### Procedure

The calibration study used a within-participant design, with each participant hearing 190 sentences. Of those, 70 were SR sentences and 70 were OR sentences, plus 50 filler sentences. No participant heard the same base sentence in both its SR and OR forms, with sentences and structures counterbalanced across participants, such that, after all participants had been tested, each base sentence had been heard in its SR and OR forms. Participants received 19 sentences at each of the compression ratios (7 SR sentences and 7 OR sentences, plus 5 filler sentences).

Instructions in all cases were to listen carefully to the sentence and, when it finished, attempt to recall the sentence as accurately and completely as possible. Speech rates and sentence types were intermixed in presentation. Stimuli were presented binaurally at 65 dB HL (hearing level) over Eartone 3A insert earphones (E-A-R Auditory systems, Aero Company, Indianapolis, IN). Prior to the experiment, participants repeated two-syllable common nouns presented at 65 dB HL to confirm audibility. This was followed by a two-part familiarization and practice session. In the first part, participants were instructed to listen to a multi-sentence passage that had been compressed to each of the compression ratios used in the present study to familiarize them with the rate of the materials and the speaker’s voice. In the second part, participants were asked to listen to and repeat one sentence at the various compression ratios. For practice for the main pupillometry experiment there were two sentences presented at a compression ratio of 100%, one sentence at both 25 and 35%, and one sentence presented at 10%. None of the familiarization stimuli were used in the study.

### Main experiment

#### Pupillary responses at critical regions of the performance curve

The main experiment measured pupil dilation to assess the level of effort participants committed to task performance. To increase power for the pupillometric portion of the study, four of the 10 compression ratios tested during the calibration study were selected to sample critical regions in the performance curve (10, 25, 35, and 100%). These ratios were chosen to sample points in the low, medium, and high range of performance to capture the hypothesized “inverted-U” pattern.

#### Participants

The participants were 32 young adults, 12 males and 20 females, aged 18–26 years. (*M* = 19.69, SD = 1.62). All had age-normal hearing, with a mean PTA of 7.6 dB HL (SD = 4.59). None of the participants from the calibration study served in the main experiment. All participants in the main experiment reported normal or corrected-to-normal vision. All participants reported English as their first language. Written informed consent was obtained from a protocol approved by the Brandeis University IRB.

#### Stimuli

The stimuli consisted of 142 SR sentences, 142 OR sentences. and 52 filler sentences as described in the calibration study. Two additional SR, OR, and filler sentences with the same characteristics were added to aid counterbalancing. Each of the SR sentences, its OR version, and the 52 filler sentences were time-compressed to 10, 25, 35 and 100% of the original playing time. The same male speaker of American English, recording procedures, and time-compression methods were as described for the calibration study.

#### Procedures

Each participant heard 192 sentences. Of those, 72 were SR sentences and 72 were OR sentences, plus the 52 filler sentences. Eighteen SR, 18 OR, and 13 filler sentences were presented at each of the four compression ratios. No participant heard the same base sentence in both its SR and OR forms, with sentences and structures counterbalanced across participants, such that, after all participants had been tested, each base sentence had been heard in its SR and OR forms and compression ratio an equal number of times. Sentence types and compression ratios were intermixed in presentation. To mitigate the potential impact of fatigue on the pupillometry data, SR and OR and speech rate trials were evenly distributed throughout the duration of the experiment. As in the calibration study, participants were instructed to listen to each sentence and then recall the sentence as accurately and completely as possible. Stimuli were presented binaurally over EAR insert earphones at 65 dB HL. The duration of the entire procedure was approximately between one and one and a half hours.

#### Pupillometry

Relative pupil dilation was measured as an index of processing effort associated with effects of syntax and compression ratio. Monocular pupil size was measured via an Eyelink 1,000 Plus eye-tracker (SR Research, Mississauga, Ontario, Canada) using a standard nine-point calibration procedure using a sampling rate of 1,000 Hz. The Eyelink camera was positioned 60 cm, zero azimuth, from the participants’ eyes, with a chinrest used to minimize participants’ head movements and to maintain the 60 cm distance between the participant’s eye and the Eye-Link camera. To reduce gaze position-dependent measurement errors (Gagl et al., 2011), eye movements were minimized by instructing participants to keep their eyes fixated on a continuously displayed 2 cm^2^ black cross centrally located on a computer screen placed above the EyeLink camera.

The computer screen was filled with a medium gray color to avoid ceiling or floor effects on the pupil size at baseline (Winn et al., 2018). Eye blinks were detected and removed using GazeR functions (Geller et al., 2020). Trials in which more than 40% of the data were missing due to blinks were removed from analyses, resulting in the exclusion of 9.07% of the trials (cf., Burg et al., 2021). Bink interpolation and signal filtering were not performed due to concerns of increasing type 1 error. The pupillary response for each sentence was baseline corrected to account for non-task changes in pupil size as can occur across trials. This was accomplished by subtracting the mean pupil size over the last 1-s of a 3-s silent period that preceded each sentence from the task-related pupil size measures (see Reilly et al., 2019, for data and a discussion of linear versus proportional baseline scaling). Although not without debate (e.g., McLaughlin et al., 2022), in cases of comparative populations or individual differences, pupil size is often normalized with respect to the pupillometric response to light range by varying screen luminance. In the present case this method was not used as the focus was within subject comparisons. Pupillary responses were aligned to the offset of each sentence.

Statistical analyses were based on the average pupil size of the final 2.5 s within a 3-s window that followed the sentence presentation. This was used to capture pupil dilation as the participant was processing the sentence and preparing their response, which is known to be sensitive to effects of cognitive effort (cf., O’Leary et al., 2025c; Piquado et al., 2010). Importantly, this region is less impacted by the potential temporal confound of stimulus length that is introduced by the use of time-compression.

#### Statistical analysis

Performance data from both the calibration and main studies, as well as pupillary responses from the main study, were analyzed using linear mixed-effects models in R version 4.3.1 (LMEM’s; Bates et al., 2015) with participants and items/stimuli included as random intercepts. We note that the items/stimuli term refers to the individual sentence items used in the experiment, not the experimental conditions. These random intercept terms were included to account for any idiosyncratic baseline difference among sentence items or participants that may be independent of the manipulated factors. For the calibration study, Compression ratios (ordered factor: 10, 15, 20, 25, 30, 35, 40, 60, 80, and 100%), Sentence type (SR vs. OR), and their interaction were included as fixed effects. The main study had the same structure of fixed and random effects, except Compression ratio had a reduced number of levels (10, 25, 35 and 100%). In all cases, a reverse selection approach was used to analyze models, starting with a maximal model including all variables and interactions. From the maximal model, likelihood ratio tests were used to contrast models and remove nonsignificant effects to find the most parsimonious model (Bates et al., 2015). Reported *p*-values are the result of likelihood ratio tests.

## Results

**Figure 2:**
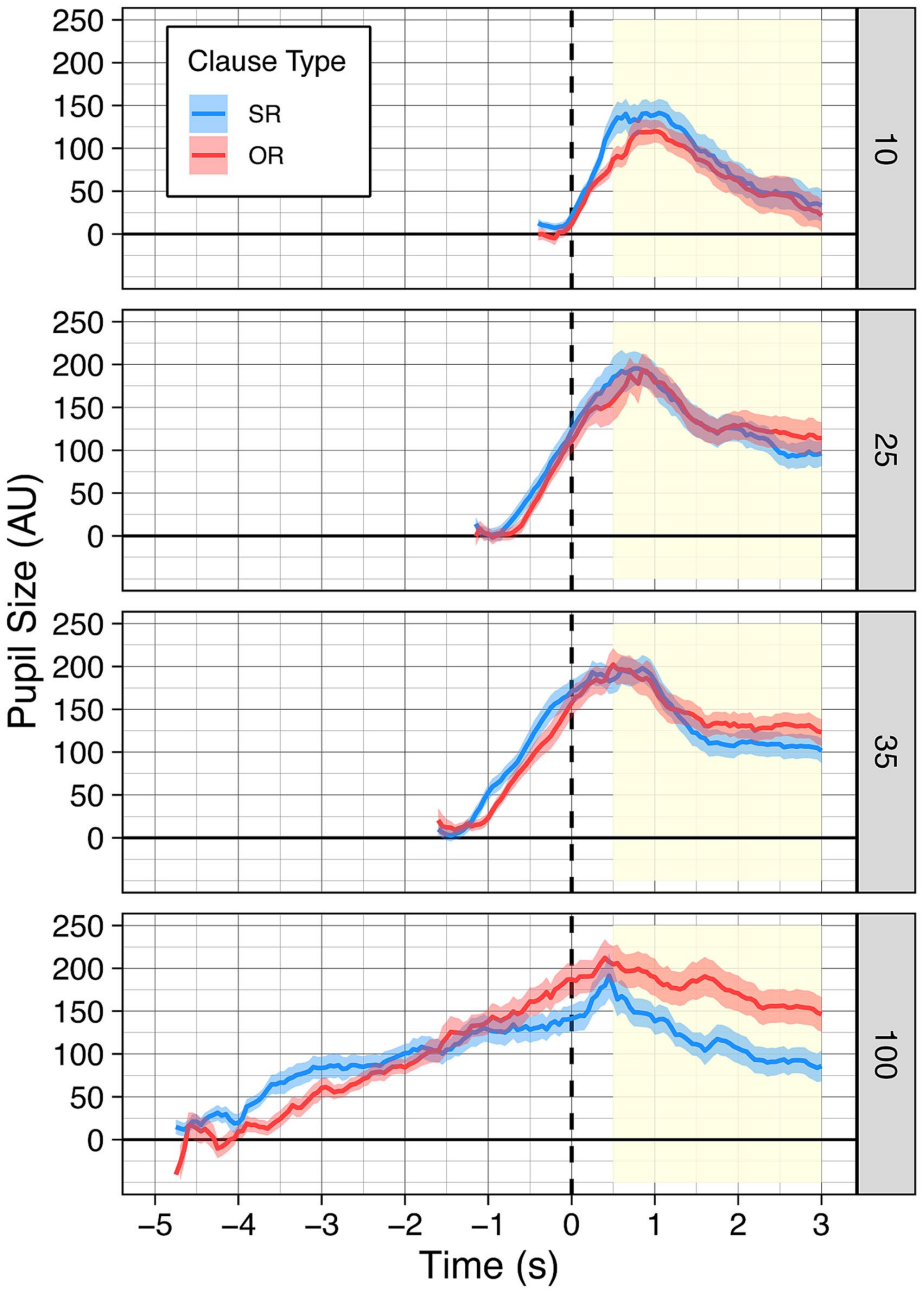
Time series of pupillometry data for sentences that were heard at each of the four compression ratios (separated by panel) for both SR (blue) and OR (red) sentences. Sentences are aligned to stimulus offset. The post-sentence region used to extract mean pupil dilation for analyses is indicated by shading in yellow. Error ribbons are one standard error.

Figure 2 shows the time series pupillary data for the SR and OR sentences for four speech rates selected on the basis of the results of the calibration on study to be described. One sees the traditional incremental increase in pupil size as the words of the sentences unfold in time. Pupil size is also shown for 3 s following the sentence presentation.

**Figure 3:**
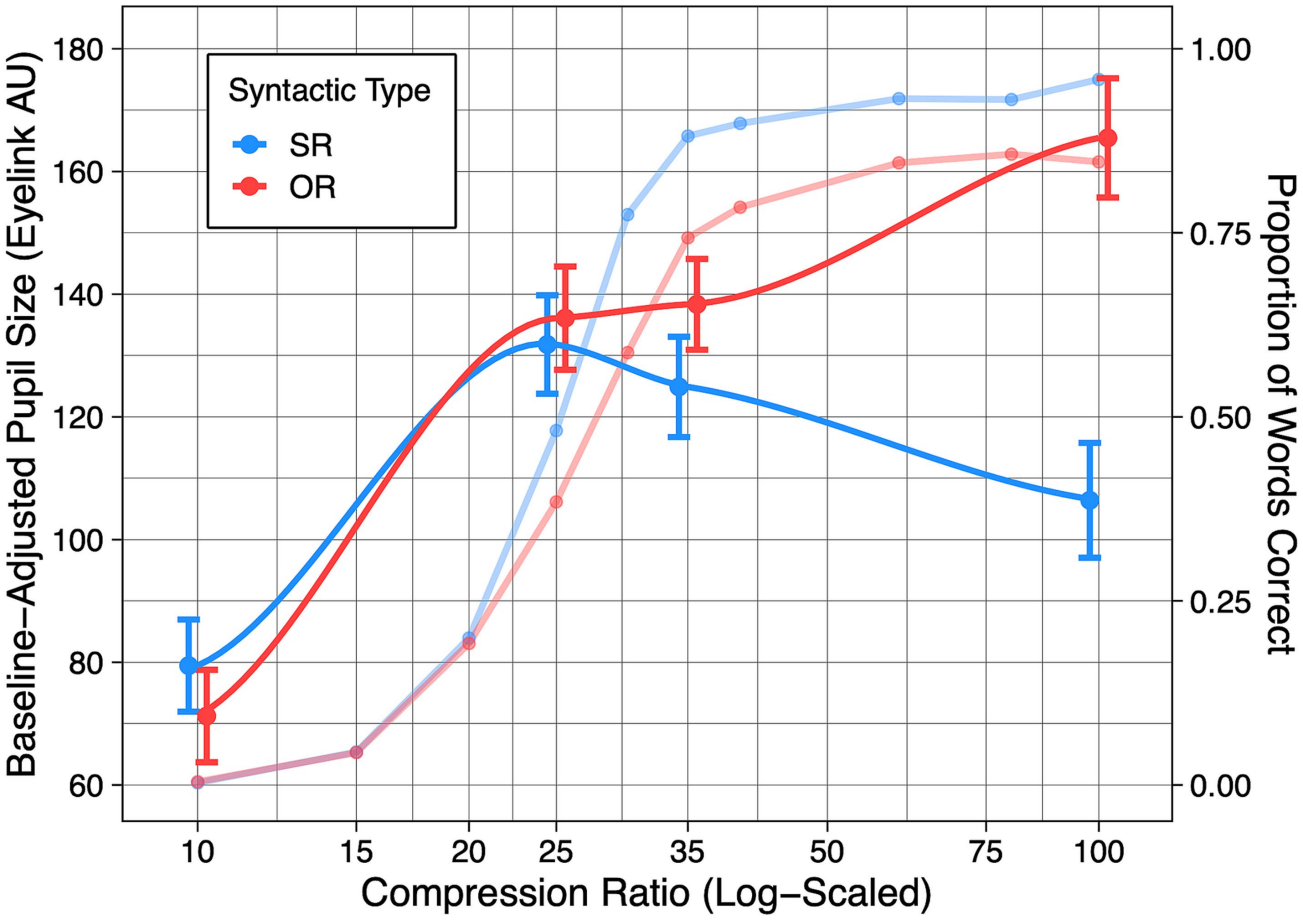
The dotted lines plot recall accuracy for SR (red) an OR (blue) sentences as a function of compression ratio based on results from the calibration study. Superimposed on these psychometric functions are the mean baseline-adjusted pupillary response during the post sentence interest regions for each of four compression ratios tested for both SR (red curve) and OR (blue curve) sentences. Error bars around the mean pupil sizes are one standard error.

Figure 3 shows two sets of curves. The two psychometric functions in Figure 3 show the mean proportion of words correctly recalled from SR and OR sentences at each of the ten compression ratios tested in the calibration study. As would be expected, recall performance yields a sinusoidal function (a progressive increase in performance accuracy with two inflection points), with recall accuracy ranging from a data-limited process when speech was heard at 10% of its original duration, rising to an asymptotic level of performance as the compression ratio was reduced (fixed effect of time-compression: χ^2^(9) = 2398.90, *p* < 0.001).

Although the shape of the functions for SR and OR sentences was similar, a distinction between SR and OR accuracy began to appear when 50% accuracy was achieved, with SR sentences reaching a performance asymptote at a higher level of accuracy than the OR sentences. This pattern was reflected by a significant main effect of sentence type (χ^2^(1) = 85.22, *p* < 0.001) and a significant Compression ratio X Sentence type interaction (χ^2^(9) = 47.77, *p* < 0.001).

Recall accuracy for the four selected compression ratios were similar to those obtained at the same compression ratios in the calibration study (see Supplementary Figure S1). Consistent with the calibration study, there was a significant effect of Compression ratio (*χ^2^*(3) = 2664.5, *p* < 0.001), a significant effect of Sentence type (*χ^2^*(1) = 95.85, *p* < 0.001), and a significant Compression ratio X Sentence type interaction (*χ^2^*(3) = 38.32, *p* < 0.001).

Figure 3 shows the mean pupillary response from the post-sentence region of interest for the SR and OR sentences at the four selected compression ratios superimposed on the performance curves from the calibration study. To visualize the inverted U-shaped pupillary response, the four sampled points were connected using the geom_smooth function of ggplot2 (Wickham, 2016). We note that, as there are only four points sampled, the smooth lines should be interpreted with caution as there are many points along the continuum that were not sampled. Results of the linear mixed effects model comparison procedure confirmed a significant main effect of Compression ratio on pupil size (*χ^2^*(3) = 85.78, *p* < 0.001), indicating that pupil size changed significantly across compression ratios. The main effect of Sentence type on pupil size was also significant (*χ^2^*(1) = 9.40, *p* = 0.002), but a significant interaction between Compression ratio and Sentence type (*χ^2^*(3) = 22.31, *p* < 0.001) indicated that the effect of syntax was not uniform at all levels of time compression. This interaction reflects the separation in pupil size between SR and OR sentences, with only the SR sentences showing the often-cited inverted U. Follow up paired t-tests were conducted at each of the compression ratios to confirm the location of this effect. As expected, mean pupil size between SR and OR sentences were not significantly different at speech rates of 10% (*t*(31) = 1.32, *p* = 0.196), 25% (*t*(31) = 0.28, *p* = 0.783), or 35% (*t*(31) = 1.22, *p* = 0.230). Supporting the interpretation that a difference in pupil size emerges between SR and OR sentences when they are heard at normal rate, the paired contrast between pupil size for SR and OR sentences at the compression rate of 100% was statistically significant (*t*(31) = 3.90, *p* < 0.001).

## Discussion

The calibration study yielded the expected sigmoidal performance functions across 10 compression ratios. From the psychometric functions, four regions of interest were sampled for the main pupillometry experiment. Recall accuracy in the main experiment closely matched the recall performance at these points in the calibration study, confirming the selection of these four compression ratios for pupillometric examination.

An equivalent floor effect in recall performance for both subject relative (SR) and object-relative (OR) sentences when speech was compressed to 10% of its normal rate followed the definition of a data-limited condition (Norman and Bobrow, 1975). The small size of the pupillary response at this point offers independent evidence of participants’ minimal commitment of effort to what was essentially an impossible task. To the extent that the size of the pupillary response is a valid index of effort, the minimal pupillary response is consistent with arguments that listeners will invest effort only to the extent that they believe this investment is likely to yield a return on this investment (Eckert et al., 2016; see also Westbrook and Braver, 2015). As postulated by Kahneman (1973), and as applied specifically to degraded speech in FUEL (Pichora-Fuller et al., 2016), there is thus a motivational element to the commitment of effort (see, for example, motivation intensity theory; Brehm and Self, 1989; Gendolla and Richter, 2013; Richter, 2013; see also Aston-Jones and Cohen, 2005).

Decreasing degrees of compression corresponding to slower speech rates resulted in a region in which an additional commitment of effort begins to bring returns; the region in which we observed an increase in mean pupil dilation accompanied by a monotonic increase in recall performance. This progressive increase in pupil dilation and performance held in a similar manner for both SR and OR sentences. When performance exceeded a level of approximately 50% correct recall for the SR sentences, a figure close to that reported by Wendt et al. (2018), pupillary responses began a decrease, reflecting the reduced effort needed as processing the slower speech required fewer resource demands. This pattern yielded the inverted U-shaped function as described in past studies (Ohlenforst et al., 2017, 2018; Wendt et al., 2018) and as appears in literature reviews (e.g., Keur-Huizinga et al., 2024; Kuchinsky and Milvae, 2023).

A different picture appears for the syntactically complex OR sentences. That is, even with a normal speech rate and asymptotic performance, mean pupil dilation fails to drop to a level approximating that for the data-limited condition that would complete the inverted U. This suggests that even though perceptual processing has become less demanding in terms of speech rate, one is still left with the resource demands associated with processing syntactically complex OR sentences (e.g., Carpenter et al., 1994; DeCaro et al., 2016).

In the time series data for the uncompressed condition, we note that differences seem to arise between the OR and SR sentence before the sentence has been completed. This pattern is not unexpected given the noncanonical word order of OR sentences. The early appearance of this sensitivity in the uncompressed condition can be attributed to the clarity of the acoustic signal and the unfolding of words at a natural rate such that listeners had sufficient time to follow the added syntactic complexity of the sentence and integrate linguistic content as it is heard, albiet at the cost of additional effort. Although likely, we note that pupillary differences within the sentences should be interpreted with caution due to the substantial variability of sentence rates, and the physiological delay of the pupillary response.

There are several accounts of why processing OR sentences is more demanding than for OR sentences, including violated agency expectations (Warren and Gibson, 2002), to the rarity of such noncanonical sentences in everyday conversation (Gibson et al., 2013; Goldman-Eisler, 1968), and to the heavier working-memory demands imposed by OR sentences compared with their SR counterparts (Cooke et al., 2002). An important finding in the present study is that the effect of syntactic difficulty is most apparent when the speech signal is clear and presented at a natural speech rate. The finding that differences in effort between OR and SR sentences appear predominantly in high signal quality conditions is consistent with the Data-Resource-Language (DRL) framework proposed by Mattys et al. (2025). The DRL framework postulates a scaffolding of operations that shift from perceptual to cognitive to linguistic as signal quality is increased. When signal quality is high, listeners have access to the highest degree of linguistic information in the sentence, and thus challenges imposed by the linguistic difficulty are reliably reflected in cognitive effort measurements.

In this study, signal quality was manipulated time-compression, which is one of many methods to perturb the speech signal. Future studies should confirm that the pattern of cognitive effort observed here generalizes to paradigms using background noise, competing speech, or noise-band vocoding. Additionally, extending this work to listeners with varying degrees of hearing loss, as well as users of cochlear implants, would help determine how individual differences in auditory perception modulates the interaction between syntactic complexity and listening effort.

We conclude that the symmetrical inverted U-shaped function in the pupillary response is limited to syntactically simple sentences, such as the 5-word active declarative sentences used in its previous demonstrations. In support of this conclusion, it can be seen that SR sentences, which are less complex than the OR sentences, but more complex than the sentences used in the demonstrations of a symmetrical inverted U, failed to yield a reduction in the pupillary response at asymptote to the level observed for the data-limited condition. With the present data we offer a more complete picture of the dynamic relationship between cognitive effort, task difficulty, and sentence complexity than currently represented in the literature.

## Data Availability

The raw data supporting the conclusions of this article will be made available by the authors, without undue reservation.
