# Supplementary material for: The “inverted-U” of cognitive effort across speech rates holds for simple but not complex sentence structures
Source: Front Psychol. 2025 Oct 29;16:1685938. doi: 10.3389/fpsyg.2025.1685938 (PMC12614458; doi:10.3389/fpsyg.2025.1685938)
Supplement: Supplementary file 1 [file Table_1.DOCX]

Supplemental Materials.

**Supplemental Figure 1.** Results for behavioral recall accuracy for SR (blue) and OR (red) sentences from the primary experiment overlayed with the psychophysical function derived from the calibration study. Error bars shown for the primary experiment are one standard error.
